# Research on the Localization Method of Outdoor Ground Vibration Signals Based on MEMS Accelerometers

**DOI:** 10.3390/s25185776

**Published:** 2025-09-16

**Authors:** Runping Liu, Xiuyan Zhao, Bin Zhou, Qi Wei

**Affiliations:** 1 School of Information Science and Engineering, Shandong Agricultural University, Tai’an 271018, China; liurunpingpp@163.com; 2 Department of Precision Instrument, Tsinghua University, Beijing 100084, China; zhoub@mail.tsinghua.edu.cn (B.Z.); weiqi@tsinghua.edu.cn (Q.W.)

**Keywords:** MEMS accelerometer, outdoor localization, ground vibration signal, TDOA localization, time delay estimation algorithm, localization algorithm, optimization algorithm

## Abstract

Addressing the need for intrusion detection and localization in critical areas, this study develops a method for outdoor ground vibration source localization utilizing subterranean-deployed MEMS accelerometers. First, the Particle Swarm Optimization (PSO) algorithm is employed to minimize the Geometric Dilution of Precision (GDOP), thereby determining the optimal configuration of the sensor array. The acquired signals are then filtered, and a novel time delay estimation algorithm, termed the Sliding Window Derivative (SWD) algorithm, is proposed. This method utilizes a sliding window to compute the sum of squared differences between adjacent sampling points within the window, generating a time-windowed energy change signal. The derivative of this signal yields a rate-of-change curve, highlighting abrupt signal transitions. The SWD algorithm, in conjunction with the STA/LTA–AIC algorithm, precisely identifies the first arrival point of the vibration signal, determining its time of arrival at each of the four sensors. Finally, an improved two-step weighted least squares method based on Time Difference of Arrival (TDOA) is used to calculate the position of the vibration source. Experimental results demonstrate an average positional error of 0.095 m and an average directional error of 0.935 degrees, validating the efficacy of the proposed method in achieving high-precision localization in outdoor environments.

## 1. Introduction

Intrusion detection and localization are critical for perimeter security in restricted and high-value areas [1,2]. However, traditional security systems face significant challenges in scenarios demanding high concealment, resilience against the electronic warfare, or complex terrain, including self-revealing signatures, vulnerability to interference or deception, and limited all-weather monitoring capabilities [3,4]. Existing localization technologies exhibit inherent limitations: video and infrared surveillance suffer from lighting and line-of-sight constraints; GPS is ineffective in indoor or subterranean environments and susceptible to jamming; and radar-based systems risk detectable electromagnetic emissions. These constraints necessitate the development of covert, robust, and high-precision localization systems, particularly in complex outdoor environments.

Localization technology is mainly divided into active localization and passive localization [5]. Active localization (such as radar, laser, etc.) relies on equipment to emit signals, and although it has high accuracy, it carries exposure risks and is susceptible to electronic interference and anti-radiation attacks. Passive localization achieves covert detection by receiving the target’s own signals (such as vibration, acoustic waves, etc.) [6]. Vibration sensors have become an ideal device for indoor and outdoor passive localization due to their concealment (subterranean deployment capability), interference immunity (not affected by lighting and Doppler effects), and low-cost advantages. Researchers and industries are also shifting their focus to low-power microelectromechanical (MEMS) sensors to replace systems based on visual devices in various real-world applications. These applications include perimeter security, healthcare, agricultural protection, wildlife tracking, and environmental monitoring [7].

The localization of ground vibration signals using MEMS vibration sensors confronts multiple challenges [8]. The interference of environmental noise such as wind and rain can overwhelm target signals. The reflection and scattering of surface waves can cause waveform distortion, resulting in inaccurate time delay estimation. Heterogeneities in soil media induce wave velocity variations, thereby compromising distance resolution.

The spatial configuration of sensors critically influences localization accuracy [9]. At present, the geometric dilution of precision (GDOP) is widely adopted as a metric to quantify the impact of sensor array geometry on localization precision. Casanova et al. [10] used evolutionary algorithms to find the geometric structure with the optimal GDOP in a certain number of base stations. Miao et al. [11] proposed using the DBSS method to dynamically select localization base stations when locating targets in monitoring areas, with GDOP as the optimization objective. This study minimizes GDOP through Particle Swarm Optimization (PSO) to derive the optimal sensor geometry configuration.

At present, target localization algorithms mainly use the following five physical measurement methods: Time of Arrival (TOA)-based localization method, Time Difference of Arrival (TDOA)-based localization method, Received Signal Strength Indicator (RSSI)-based localization method, Angle of Arrival (AOA)-based localization method, and Direction of Arrival (DOA)-based localization method [12]. Among them, the TOA localization algorithm requires high-precision time synchronization between the transmitting and receiving ends, which is difficult to achieve in practice and can result in significant errors; the RSSI localization algorithm is greatly affected by the environment and has low localization accuracy; the AOA localization algorithm requires the use of antenna arrays or phased arrays to measure the angle at which signals arrive, which cannot meet the requirements of this scenario and is more suitable for sound localization; DOA is similar to AOA and does not meet the requirements of this scenario [13]. The TDOA localization algorithm has lower requirements for the above, and performs well in high-precision and high robustness systems. Therefore, TDOA is adopted as the core localization framework.

In the physical measurement methods using TDOA, the first and most important thing is to find a suitable time-delay estimation algorithm, and several scholars have conducted research in this area. Shi et al. [14] proposed a time-delay estimation method based on volume cross-correlation function, which utilizes a subspace linear correlation and a volume metric to achieve super-resolution time-delay estimation under signal conditions. Lellouch et al. [15] proposed using cross-correlation Hilbert envelope calculation for time-delay estimation, combined with multi band filtering preprocessing and consistency verification to achieve sound source localization in a four microphones array. Li et al. [16] proposed using a trilinear decomposition based on the PARAFAC model to estimate the delay matrix, and then extracting the time-delay estimate using the structural characteristics and differential operations of the matrix. Zhu et al. [17] proposed using an improved cross-correlation function to effectively suppress TDOA estimation errors under low signal-to-noise ratio through dual optimization of probability statistical filtering and physical constraint window. The above method significantly reduces the accuracy of time-delay estimation in outdoor low signal-to-noise ratio vibration signals due to reflection in the propagation path and other reasons. This article intends to propose a new delay estimation algorithm to overcome this bottleneck. Secondly, after obtaining the time-delay estimation, an appropriate localization algorithm can calculate the precise target position. Heider et al. [18] proposed using the STA/LTA algorithm to select time windows, calculating time-delay estimation through cross-correlation, and determining the location of footprints through grid search, achieving localization results with a deviation of less than one meter within an outdoor range of 30 m × 30 m. Choudhary et al. [19] proposed a method of using seismic sensors in outdoor environments and utilizing the regression and characteristics of seismic waves for localization, with an average localization error of 1.37 m. Choudhary et al. [20] proposed a regression based approach that utilizes seismic and audio features for personnel localization, achieving root mean square localization errors of 0.735 m and 0.907 m for two datasets, respectively. Drira et al. [21] proposed a model-based method using EDMF to overcome the fuzzy interpretation of vibration measurement caused by obstacles and different floor stiffness, achieving a 50% improvement in localization accuracy. Chiasson et al. [22] proposed UWB localization technology based on asynchronous hyperbolic localization, which eliminates clock synchronization requirements through the TDOA equation and achieves a positional error of 6.9 cm for two-dimensional source localization. Lakshmi et al. [23] proposed a TDOA measurement model using RSSI and the Chan algorithm to determine the coordinates of unknown nodes, achieving a distance value of positional error close to 2.7 cm. Among the different localization algorithms mentioned above, the outdoor localization accuracy is poor, and indoor localization cannot be used due to significant differences in wave propagation media between indoor and outdoor localization. This article adopts an improved two-step weighted least squares algorithm that integrates residual weighting to suppress the propagation of outdoor environmental errors.

## 2. Vibration Wave Propagation Model

When vibration signals propagate in a medium, various types of vibration waves are formed. According to the vibration characteristics of the medium and the propagation mechanism of the waves, these waves are usually divided into two categories: body waves and surface waves [24]. Body waves include Primary waves (P waves) and Shear waves (S waves). The propagation direction of P waves is parallel to the vibration direction of the medium particles, which belongs to volumetric strain. The propagation direction of S waves is perpendicular to the vibration direction of the medium particles and belongs to shear strain [25]. On the surface of the Earth, P waves and S waves typically propagate independently of each other, but may experience reflection or refraction during wavefront interactions.

Surface waves mainly propagate along the boundaries of the medium and can be converted from body waves. The energy of such waves is most concentrated at the interface and decays exponentially with increasing depth [26]. Surface waves can be further divided into Love waves (L waves) and Rayleigh waves (R waves). The propagation conditions of L waves are relatively limited. The direction of motion of the medium particles is both perpendicular to the direction of wave propagation and parallel to the surface of the medium, and the propagation speed is close to that of S waves, which makes it difficult for sensors to detect them [27]. In contrast, R waves propagate along the surface of the medium, and the trajectory of particle motion appears elliptical, with a slightly lower propagation speed than S waves [28].

**Figure 1:**
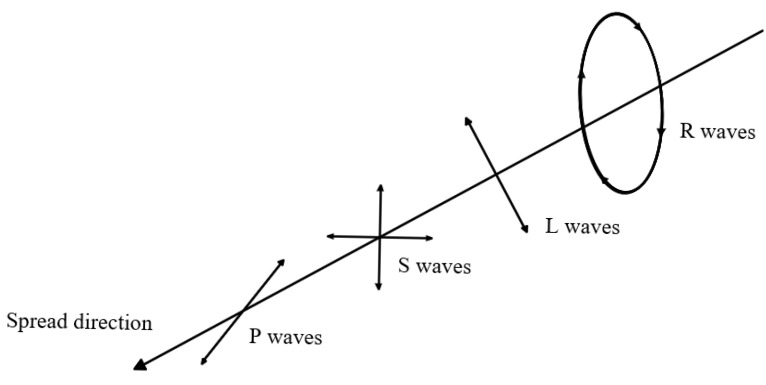
Vibration wave train.

According to the above analysis of vibration waves, the vibration wave sequences observed at positions far from the vibration source are P waves, S waves, L waves, and R waves, as shown in Figure 1.

In practical engineering surveying, P waves, S waves, and R waves are the main receiving targets of sensors [24]. However, there are significant differences in the propagation characteristics of these three waves: P-waves have the fastest propagation speed and higher frequency, making it difficult to accurately capture their signals in measurements; Although the propagation speed of S wave is lower than that of P wave, its energy is relatively small, and the signal characteristics are not easy to distinguish clearly; R waves, as waves propagating along the surface of a medium, have a relatively low velocity, the strongest energy, and the main frequency is concentrated around 150 Hz [29]. Therefore, it is often prioritized as the receiving and analyzing object in practical measurements.

## 3. Sensor Deployment Configuration

### 3.1. GDOP

The geometric configuration of sensors is an important factor affecting the accuracy of localization. The localization performance largely depends on the geometric position relationship between the target to be measured and the sensor. Different deployment configurations of sensors have different localization accuracies for targets in the same area. The geometric accuracy factor (GDOP) is a key indicator for measuring the degree to which localization accuracy is affected by geometric relationships. For unbiased estimation systems, the expression of the geometric accuracy factor GDOP is

(1) $$GDOP=\sqrt{tr\left[{\left(A^{T}A\right)}^{-1}\right]}\;$$

where tr(·) represents the transpose of the matrix, A is a coefficient matrix in the localization equation system, and in a two-dimensional coordinate system, GDOP can also be expressed as

(2) $$GDOP=\sqrt{\sigma_{x}^{2}+\sigma_{y}^{2}}\;$$

where the square of the localization standard deviation in the two directions of *x* and *y* is represented by $\sigma_{x}^{2}$ and $\sigma_{y}^{2}$, respectively. The measurement of the influence of the geometric configuration of the sensor on localization accuracy is usually based on GDOP as a design indicator. The smaller the overall GDOP, the more reasonable the sensor layout.

### 3.2. Particle Swarm Optimization

Particle Swarm Optimization (PSO) [30] algorithm is a swarm-intelligence-based optimization algorithm proposed by James Kennedy and Russell Eberhart in 1995. This algorithm originated from the study of the hunting behavior of bird flocks. Similarly to genetic algorithm, it iteratively searches for the optimal solution. The basic principles involve updating the position and velocity of particles, as well as individual and social learning.

The PSO algorithm uses each particle to search for the optimal value in the solution space. Each particle has two attributes: position and velocity. Position represents potential solutions, while velocity determines its search direction and stride. The initialization and velocity update of particles are shown in Equations (3) and (4):

(3) $$x_{ij}=rand\cdot \left(UB_{j}-LB_{j}\right)+LB_{j}\;i=1,2\ldots ,N,\;j=1,2,\ldots ,n$$

(4) $$v_{ij}\left(t+1\right)=\omega v_{ij}\left(t\right)+c_{1}r_{1}\left(t\right)\left(p_{ij}\left(t\right)-x_{ij}\left(t\right)\right)+c_{2}r_{2}\left(t\right)\left(p_{gj}\left(t\right)-x_{ij}\left(t\right)\right)$$

where rand is a random number between 0 and 1, $LB_{j}$ represents the jth lower limit value of the given problem, and $UB_{j}$ represents the jth upper limit value of the given problem. $v_{ij}\left(t\right)$ is the velocity of particle i at time t in dimension j, $x_{ij}\left(t\right)$ is the current position, $p_{ij}\left(t\right)$ is the best position of the particle so far, $p_{gj}\left(t\right)$ is the global best position, ω is the inertia weight, $c_{1}$ and $c_{2}$ are learning factors, and $r_{1}\left(t\right)$ and $r_{2}\left(t\right)$ are random numbers in the [0,1] interval. The update of particle motion position is shown in Equation (5):

(5) $$x_{ij}\left(t+1\right)=x_{ij}\left(t\right)+v_{ij}\left(t+1\right)$$

The main advantage of PSO algorithm lies in its simplicity and ease to implement characteristics, and it also has fast convergence speed. This makes PSO a powerful tool to solve complex optimization problems.

### 3.3. Geometric Configuration Optimization Based on PSO

By minimizing the GDOP in a certain range, the optimal geometric configuration of the sensor can be obtained. The specific steps are as follows:

(1) The GDOP value is calculated by taking the edge distance of 0.5–5 m, respectively, to determine the general range of the optimal geometric configuration.
(2) Select the interval with small GDOP as the optimization range, and initialize parameters such as population size and iteration times. The coordinates of the four sensors are optimized output parameters. Where $X\in \;\left[0,4\right],\;Y\in \;\left[-4,0\right]$.
(3) Select the fitness function. In order to find the geometric configuration with minimal GDOP, the GDOP value within the configuration range is taken as the fitness function.
(4) The accuracy calculation is performed and the position of the solution is updated according to the weight allocation strategy.
(5) Update and record the location of the solution.
(6) Based on the current minimum GDOP value, the fitness is updated and the location of the optimal solution is determined.
(7) By repeating steps (4) to (6), when the maximum number of iterations is reached, the position of the optimal solution is output, and the best combination of X and Y under the current data conditions is determined.

## 4. Delay Estimation Algorithm

### 4.1. Traditional Time Delay Estimation Algorithm

#### 4.1.1. Cross Correlation

As a classic algorithm of TDOA localization system, cross-correlation time delay estimation realizes time delay detection by quantifying the similarity of two signals. Suppose $x_{1}\left(t\right)$ and $x_{2}\left(t\right)$ are the signals from the vibration source $s\left(t\right)$ to the two sensors, respectively, as shown in Equation (6):

(6) $$\begin{array}{c} x_{1}\left(t\right)=a_{1}s\left(t-t_{1}\right)+n_{1}\left(t\right)\\ x_{2}\left(t\right)=a_{2}s\left(t-t_{2}\right)+n_{2}\left(t\right) \end{array}$$

where $a_{1}$ and $a_{2}$ are the amplitude loss factors of the two signals in their respective channel propagation. $t_{1}$ and $t_{2}$ are the time when the vibration source $s\left(t\right)$ reaches different sensors, respectively. $n_{1}\left(t\right)$ and $n_{2}\left(t\right)$ are the noise interference values received by the vibration source $s\left(t\right)$ in their respective channels. Assuming that the signal and noise in the model are independent and independent of each other, then Equation (7) can be obtained:

(7) $$\begin{array}{l} x_{1}\left(t\right)=s\left(t\right)+n_{1}\left(t\right)\;\\ x_{2}\left(t\right)=as\left(t-T\right)+n_{2}\left(t\right) \end{array}$$

where a is the normalized amplitude loss factor and $T=\left|t_{1}-t_{2}\right|$ is the time delay. If the loss factor is 1, the cross-correlation function of $x_{1}\left(t\right)$ and $x_{2}\left(t\right)$ is calculated as

(8) $$R_{x1x2}\left(\tau \right)=R_{ss}\left(\tau -T\right)$$

The autocorrelation function has the following properties:

(9) $$\left|R_{ss}\left(\tau -T\right)\right|\leq R_{ss}\left(0\right)$$

According to this property, when $\left(\tau -T\right)=0$, the value of autocorrelation function is the largest, indicating that the correlation degree between the two signals is the highest. Thus, when the autocorrelation function reaches the maximum value τ−T, it is the estimated value of time delay between two signals.

#### 4.1.2. STA/LTA

In addition to the time-delay estimation method based on cross-correlation, the time-delay estimation between sensors can also be extracted by the first arrival time method of elastic wave. For example, STA/LTA, which is a classic method for first break detection of seismic signals, calculates the energy of each window on the time axis by using the difference in amplitude between vibration signals and noise signals and the sliding long and short time windows, and distinguishes the effective signal and noise signal by the ratio of energy, so as to determine the first break arrival time of vibration waves [31]. The core principle is shown in Equation (10):

(10) $$\frac{STA}{LTA\left(i\right)}\;=\;\frac{N_{LTA}\sum_{l\;=\;i\;-\;N_{STA}}^{i}CF\left(l\right)}{N_{STA}\sum_{l\;=\;i\;-{\;N}_{LTA}}^{i}CF\left(l\right)}$$

Among them, $CF\left(l\right)$ is the characteristic function of the collected data. The purpose of using the characteristic function is to magnify the difference in the collected data. The calculation operations of common characteristic functions include taking absolute value, data square and first derivative, etc.

### 4.2. SWD–STA/LTA–AIC

In order to improve the accuracy and robustness of time delay estimation, an improved STA/LTA–AIC algorithm based on sliding window derivative (SWD) is proposed in this paper. The algorithm enhances the mutation feature through signal preprocessing, which significantly improves the performance of traditional methods.

#### 4.2.1. AIC

The STA/LTA algorithm needs to manually determine the threshold, time window length and other parameters. The characteristic function can distinguish the characteristic difference between the vibration signal and the noise signal. When the characteristic function is different, its emphasis on picking up the signal is also different. In this paper, it is combined with the energy method, so the characteristic function is set as the square of the sequence amplitude. If the threshold parameter is set too high, the pickup position will be backward and the time delay will become longer. If the threshold parameter is set too low, the noise will be picked up and the time delay estimation calculation will become shorter. Therefore, STA/LTA algorithm is usually used in the first step of first break picking. After determining the approximate location of first break arrival, AIC algorithm is used to accurately locate the arrival time of first break.

The AIC value of the AR model can be expressed as

(11) $$AIC\left(k\right)=2k+Nlog\sigma_{\varepsilon }^{2}$$

where N is the number of sampling points, k is the order of AR model, and $AIC\left(k\right)$ is the likelihood of AR model.

The simplified AIC algorithm does not need to calculate the order of signal AR model, which greatly reduces the amount of calculation. The AIC value expressed by the algorithm is shown in Equation (12):

(12) $$AIC\left(k\right)=kln\left(var\left\{x\left(1,k\right)\right\}\right)+(N-k-1)ln\left(var\left\{x\left(k+1,N\right)\right\}\right)$$

where $var\left\{x\left(1,k\right)\right\}$ is the variance of the former partial sequence $x\left(1\right),x\left(2\right),\ldots ,x\left(k\right)$, and $x\left(k+1,N\right)$ is the variance of the latter partial sequence $x\left(k+1\right),x\left(k+2\right),\ldots ,x\left(N\right)$.

The algorithm uses the statistical characteristics of the vibration signal and the noise signal before arrival to separate the signal reasonably. If the AIC value is the smallest, it indicates that the fitting degree of the two parts of the signal is the worst here, which is the initial point of the effective signal. Generally, AIC algorithm can only be used when the first break position is known. When used alone, it is easy to have a local minimum in the calculation result. The time corresponding to this local minimum is not necessarily the arrival time of the real first break. The combination of STA/LTA algorithm and AIC algorithm can improve the reliability of the results, so first use the STA/LTA algorithm to determine the approximate area of the arrival time of the first break, and then calculate the point with the minimum AIC in the time window according to AIC algorithm, which is the arrival time of the first break.

#### 4.2.2. Sliding Window Derivative

Slow changes such as instrument drift and environmental low-frequency interference will affect the baseline of STA/LTA value, which may lead to false triggering or delayed triggering, thus affecting the AIC algorithm to enter the local minimum. In order to overcome the above shortcomings, this paper proposes an SWD preprocessing algorithm. The specific implementation process includes the following steps:

(1) Sliding window differential sum of squares

(13) $$E_{w}\left(t\right)=\sum_{k=t}^{t+w-1}{\left(x_{k+1}-x_{k}\right)}^{2}$$

where w is the window length, and $E_{w}\left(t\right)$ quantifies the energy change intensity of the signal in the local window.

(2) Derivative operation

(14) $$D\left(t\right)={\;E}_{w}\left(t\right)-E_{w}\left(t-1\right)$$

The derivative signal keenly captures the instantaneous state of energy change and forms the characteristic peak of the first solstice.

SWD pretreatment improves the detection performance of the first break through the dual enhancement mechanism, which can suppress random noise and low-frequency components, is insensitive to the overall energy change in the signal, and focuses on the relative mutation. The sharp pulse peak makes the peak positioning more accurate.

## 5. Implementation of TDOA Localization Algorithm

The time difference of arrival (TDOA) method is an important localization method, which determines the position of the vibration source by calculating the time delay of the vibration signal reaching multiple sensors, and has high accuracy. The following describes the principle of TDOA.

**Figure 2:**
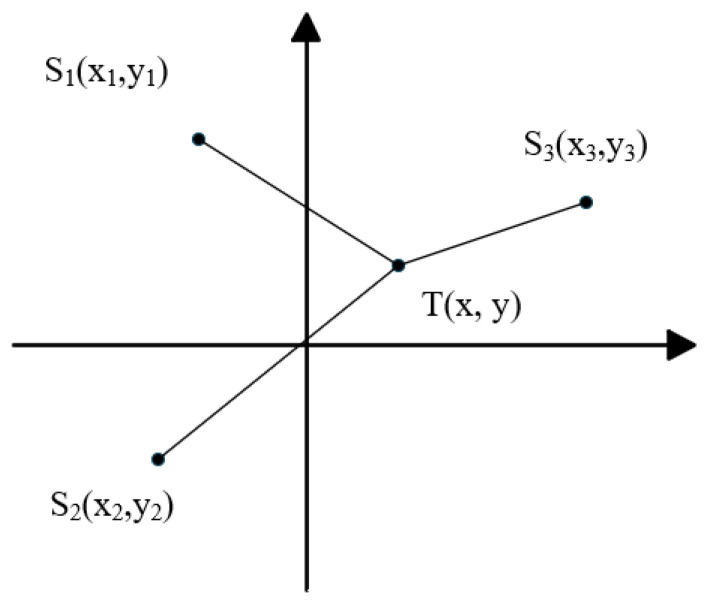
Schematic diagram of the TDOA principle.

As shown in Figure 2, T(x,y) is the position of the vibration source, and $S_{1}\left(x_{1},y_{1}\right),\;S_{2}(x_{2},y_{2})$ and $S_{3}(x_{3},y_{3})$ are the positions of the three sensors, respectively.

Suppose that the distances from the three sensors to the vibration source are $r_{1},\;r_{2}$ and $r_{3}$ respectively, the difference in distance is $∆r_{i1}$, which is the difference between $r_{i}$ and $r_{1}$, i=2, 3 and c is the vibration propagation velocity, then the position equation can be obtained:

(15) $$\left\{\begin{array}{c} r_{1}^{2}={\left(x-x_{1}\right)}^{2}+{\left(y-y_{1}\right)}^{2}\\ r_{i}^{2}={\left(x-x_{i}\right)}^{2}+{\left(y-y_{i}\right)}^{2}\\ c\cdot t_{i1}=r_{i}-r_{1}=r_{i1} \end{array}\right.$$

From Equation (15):

(16) $$r_{i}^{2}-r_{1}^{2}=x_{i}^{2}-x_{1}^{2}+y_{i}^{2}-y_{1}^{2}-2x\left(x_{i}-x_{1}\right)-2y\left(y_{i}-y_{1}\right)$$

Another reason

(17) $$r_{i}^{2}-r_{1}^{2}=r_{i1}^{2}+2r_{1}r_{i1}$$

By substituting into Equation (16), the following can be obtained:

(18) $$\frac{1}{2}[{\left(ct_{i1}\right)}^{2}+x_{i}^{2}+y_{i}^{2}-x_{1}^{2}-y_{1}^{2}]+{ct}_{i1}r_{1}=x\left(x_{1}-x_{i}\right)+y\left(y_{1}-y_{i}\right)$$

Command

(19) $$\frac{1}{2}\left[{\left(ct_{i1}\right)}^{2}+x_{i}^{2}+y_{i}^{2}-x_{1}^{2}-y_{1}^{2}\right]=k_{i}$$

Then,

(20) $$k_{i}+{ct}_{i1}r_{1}=x\left(x_{1}-x_{i}\right)+y\left(y_{1}-y_{i}\right)$$

Among them, the vibration propagation velocity c can be obtained by averaging the wave velocity at multiple known points, where $t_{i1},\;x_{1},\;y_{1},\;x_{i},\;y_{i}$ are known quantities, and $x,\;y,\;r_{1}$ are unknown quantities. By solving Equation (20), the position of the vibration target can be obtained. There are currently multiple methods available to solve this equation, such as direct solving, the Taylor algorithm, and the Chan algorithm.

### 5.1. Traditional Localization Algorithm

#### 5.1.1. Direct Solution Method

Rewrite the positioning equation of Equation (20) into matrix equation form:

(21) AX=F

where

(22) $$A=\left[\begin{array}{cc} x_{1}-x_{2} & y_{1}-y_{2}\\ x_{1}-x_{3} & y_{1}-y_{3} \end{array}\right]$$

(23) $$X=\left[\begin{array}{c} x\\ y \end{array}\right]$$

(24) $$F=\left[\begin{array}{c} k_{2}+r_{21}r_{1}\\ k_{3}+r_{31}r_{1} \end{array}\right]$$

For the three-point positioning method on the plane, select three sensor positions reasonably so that the rank of matrix A is not 1. At this time, the estimated positions are

(25) $$\hat{X}=A^{-1}F$$

where

(26) $$A^{-1}=\frac{1}{\left|A\right|}A^{*}=\frac{1}{\left(x_{1}-x_{2}\right)\left(y_{1}-y_{3}\right)-(x_{1}-x_{3})(y_{1}-y_{2})}\left[\begin{array}{cc} y_{1}-y_{3} & {-y}_{1}+y_{2}\\ {-x}_{1}+x_{3} & x_{1}-x_{2} \end{array}\right]$$

(27) $$\left[\begin{array}{c} x\\ y \end{array}\right]=\frac{\left[\begin{array}{cc} y_{1}-y_{3} & {-y}_{1}+y_{2}\\ {-x}_{1}+x_{3} & x_{1}-x_{2} \end{array}\right]\left[\begin{array}{c} k_{2}+r_{21}r_{1}\\ k_{3}+r_{31}r_{1} \end{array}\right]}{\left(x_{1}-x_{2}\right)\left(y_{1}-y_{3}\right)-(x_{1}-x_{3})(y_{1}-y_{2})}$$

It can be concluded that

(28) $$\hat{x}=\frac{\left\{\left[\left(y_{1}-y_{3}\right)k_{2}+\left({x_{3}-x}_{1}\right)k_{3}\right]+\left[\left(y_{1}-y_{3}\right)r_{21}+\left({x_{3}-x}_{1}\right)r_{31}\right]r_{1}\right\}}{\left(x_{1}-x_{2}\right)\left(y_{1}-y_{3}\right)-(x_{1}-x_{3})(y_{1}-y_{2})}$$

(29) $$\hat{y}=\frac{\left\{\left[\left({y_{2}-y}_{1}\right)k_{2}+\left(x_{1}-x_{2}\right)k_{3}\right]+\left[\left({y_{2}-y}_{1}\right)r_{21}+\left(x_{1}-x_{2}\right)r_{31}\right]r_{1}\right\}}{\left(x_{1}-x_{2}\right)\left(y_{1}-y_{3}\right)-(x_{1}-x_{3})(y_{1}-y_{2})}$$

Command

(30) $$\begin{array}{c} \begin{array}{c} m_{1}=\frac{\left(y_{1}-y_{3}\right)k_{2}+\left({x_{3}-x}_{1}\right)k_{3}}{\left(x_{1}-x_{2}\right)\left(y_{1}-y_{3}\right)-(x_{1}-x_{3})(y_{1}-y_{2})}\\ m_{2}=\frac{\left({y_{2}-y}_{1}\right)k_{2}+\left(x_{1}-x_{2}\right)k_{3}}{\left(x_{1}-x_{2}\right)\left(y_{1}-y_{3}\right)-(x_{1}-x_{3})(y_{1}-y_{2})} \end{array}\\ \begin{array}{c} n_{1}=\frac{\left(y_{1}-y_{3}\right)r_{21}+\left({x_{3}-x}_{1}\right)r_{31}}{\left(x_{1}-x_{2}\right)\left(y_{1}-y_{3}\right)-(x_{1}-x_{3})(y_{1}-y_{2})}\\ n_{2}=\frac{\left({y_{2}-y}_{1}\right)r_{21}+\left(x_{1}-x_{2}\right)r_{31}}{\left(x_{1}-x_{2}\right)\left(y_{1}-y_{3}\right)-(x_{1}-x_{3})(y_{1}-y_{2})} \end{array} \end{array}$$

Then,

(31) $$\left\{\begin{array}{c} \hat{x}=m_{1}+n_{1}r_{1}\\ \hat{y}=m_{2}+n_{2}r_{1} \end{array}\right.$$

Substituting Equation (28) into Equation (15) yields

(32) $$r_{1}^{2}={\left(m_{1}+n_{1}r_{1}-x_{1}\right)}^{2}+{\left(m_{2}+n_{2}r_{1}-y_{1}\right)}^{2}$$

Simplified,

(33) $$\left(n_{1}^{2}+n_{2}^{2}-1\right)r_{1}^{2}+2\left[\left(m_{1}-x_{1}\right)n_{1}+(m_{2}-y_{1})n_{2}\right]r_{1}+{\left(m_{1}-x_{1}\right)}^{2}+{\left(m_{2}-y_{1}\right)}^{2}=0$$

Command

(34) $$\left\{\begin{array}{c} a=\left(n_{1}^{2}+n_{2}^{2}-1\right)\\ b=\left(m_{1}-x_{1}\right)n_{1}+(m_{2}-y_{1})n_{2}\\ {\left(m_{1}-x_{1}\right)}^{2}+{\left(m_{2}-y_{1}\right)}^{2} \end{array}\right.$$

Then,

(35) $$ar_{1}^{2}+2br_{1}+c=0$$

It can be obtained using the root finding formula

(36) $$r_{1}=\frac{-b\pm \sqrt{b^{2}-4ac}}{a}$$

Equation (36) can provide two solutions, $r_{11}$ and $r_{12}$. Because they represent distance, if there are negative roots, they can be directly discarded, and the other value is the true value. But if both values are positive roots, it is necessary to arrange the position of the sensor reasonably or place an additional sensor as a reference point to make the localization more accurate.

#### 5.1.2. Taylor Series Expansion Method

The Taylor series expansion method is a recursive calculation method based on the initial position of the target. Gradually converge to the estimated position in each recursion by solving the local least squares solution of the TDOA measurement error $\left(e_{1},e_{2}\right)$. For a set of TDOA measurement values, based on the selected initial position of the target $\left(\hat{x},\hat{y}\right)$, perform the Taylor series expansion on Equation (15) at $\left(\hat{x},\hat{y}\right)$ to remove components of second order or higher. Then, Equation (15) is transformed into

(37) e≈h−Gδ

where

(38) $$e=\left[\begin{array}{c} e_{1}\\ e_{2} \end{array}\right]$$

Indicate the difference between the TDOA measurement value and the true value.

(39) $$h=\left[\begin{array}{c} c\tau_{1}-(\hat{r_{1}}-\hat{r_{0}})\\ c\tau_{2}-(\hat{r_{2}}-\hat{r_{0}}) \end{array}\right]$$

(40) $$G=\left[\begin{array}{cc} \frac{\partial ∆r_{1}}{\partial x} & \frac{\partial ∆r_{1}}{\partial y}\\ \frac{\partial ∆r_{2}}{\partial x} & \frac{\partial ∆r_{2}}{\partial y} \end{array}\right]$$

Command $x=\hat{x},y=\hat{y}$. The weighted least squares method can be used to solve the target position deviation:

(41) $$\delta =\left[\begin{array}{c} ∆x\\ ∆y \end{array}\right]={\left(G^{T}Q^{-1}G\right)}^{-1}G^{T}Q^{-1}h$$

In Equation (41), Q represents the covariance matrix of the TDOA measurement values. In the following recursive process, substitute ${\hat{x}}^{1}=\hat{x}+∆x,\;{\hat{y}}^{1}=\hat{y}+∆y$, and repeat the previous steps until δ is small enough. At this point, $\left({\hat{x}}^{1},\;{\hat{y}}^{1}\right)$ is considered to be the estimated position of the target.

The Taylor series expansion method can achieve good estimation values in most environments, but because it is a recursive algorithm, it cannot provide a clear expression solution. Moreover, the algorithm requires an initial estimated coordinate that is close to the actual coordinate to improve its convergence probability. However, this is difficult to achieve in practical applications, and it cannot make judgments on possible non convergence situations in advance. It also requires prior information about TDOA measurement values to determine the weighting matrix Q. Therefore, the computational complexity of the Taylor series expansion method is relatively large.

#### 5.1.3. Chan Algorithm

The Chan algorithm [32] is a non-recursive solution for hyperbolic equations with analytical expressions. The characteristic of this algorithm is its low computational complexity and good localization accuracy.

The basic principle of Chan algorithm is introduced below. Taking the four sensors in the plane as an example, let the positions of the four sensors be $S_{1}\left(x_{1},\;y_{1}\right),\;S_{2}\left(x_{2},\;y_{2}\right),\;S_{3}\left(x_{3},\;y_{3}\right),\;S_{4}\left(x_{4},\;y_{4}\right)$, respectively. The vibration source position is T(x, y), and the distance from the sensor to the vibration source is $r_{i}=\sqrt{{\left(x-x_{i}\right)}^{2}+{\left(y-y_{i}\right)}^{2}}$.

Therefore,

(42) $${r_{i}}^{2}={\left(x-x_{i}\right)}^{2}+{\left(y-y_{i}\right)}^{2}=k_{i}-2xx_{i}-2yy_{i}+x^{2}+y^{2}$$

(43) $${r_{1}}^{2}={\left(x-x_{1}\right)}^{2}+{\left(y-y_{1}\right)}^{2}=k_{1}-2xx_{1}-2yy_{1}+x^{2}+y^{2}$$

where $k_{i}={x_{i}}^{2}+{y_{i}}^{2}$, let $r_{i1}$ represent the distance difference between sensor $S_{i}$ and the target relative to sensor $S_{1}$, then

(44) $$r_{i1}=r_{i}-r_{1}$$

Combining Equations (42) and (43):

(45) $${r_{i}}^{2}-{r_{1}}^{2}=k_{i}-k_{1}+2x\left(x_{1}-x_{i}\right)+2y\left(y_{1}-y_{i}\right)$$

(46) $${r_{i}}^{2}-{r_{1}}^{2}={r_{i1}}^{2}+2r_{i}r_{1}-2{r_{1}}^{2}$$

Combining Equations (45) and (46):

(47) $$x\left(x_{i}-x_{1}\right)+y\left(y_{i}-y_{1}\right)+r_{1}r_{i1}=\frac{1}{2}\left(k_{i}-k_{1}-{r_{i1}}^{2}\right)$$

Construct a matrix representation:

(48) $$\left[\begin{array}{c} x\\ y \end{array}\right]=-{\left[\begin{array}{cc} x_{2}-x_{1} & y_{2}-y_{1}\\ x_{3}-x_{1} & y_{3}-y_{1}\\ x_{4}-x_{1} & y_{4}-y_{1} \end{array}\right]}^{-1}\left\{\left[\begin{array}{c} r_{21}\\ r_{31}\\ r_{41} \end{array}\right]r_{1}+\frac{1}{2}\left[\begin{array}{c} k_{2}-k_{1}-{r_{21}}^{2}\\ k_{3}-k_{1}-{r_{31}}^{2}\\ k_{4}-k_{1}-{r_{41}}^{2} \end{array}\right]\right\}$$

Substitute $r_{1}=\sqrt{{\left(x-x_{1}\right)}^{2}+{\left(y-y_{1}\right)}^{2}}$ to obtain a quadratic equation about $r_{1}$, and solve for the estimated position (x, y) of the target.

### 5.2. Improved Two-Step Weighted Least Squares Method

The improved two-step weighted least squares (ITWLS) method is an improvement based on the Chan algorithm and two-step weighted least squares method, which can further improve the accuracy of localization. The following introduces the principle of the improved two-step weighted least squares method.

Convert the linear equation established by the Chan algorithm into matrix form:

(49) Gaθ=h

where

(50) $$\begin{array}{c} \theta =\left[\begin{array}{c} x\\ y\\ r_{1} \end{array}\right]\;\\ Ga=\left[\begin{array}{ccc} x_{2}-x_{1} & y_{2}-y_{1} & r_{21}\\ x_{3}-x_{1} & y_{3}-y_{1} & r_{31}\\ x_{4}-x_{1} & y_{4}-y_{1} & r_{41} \end{array}\right]\\ h=\frac{1}{2}\left[\begin{array}{c} k_{2}-k_{1}-{r_{21}}^{2}\\ k_{3}-k_{1}-{r_{31}}^{2}\\ k_{4}-k_{1}-{r_{41}}^{2} \end{array}\right] \end{array}$$

Initialize weighted least squares estimation. Using the measurement noise covariance matrix Q:

(51) $$\theta_{0}={\left({Ga}^{T}Q^{-1}Ga\right)}^{-1}{Ga}^{T}Q^{-1}h$$

Obtain initial estimate: $\theta_{0}={\left[x_{0},\;y_{0},\;r_{1,0}\right]}^{T}$. Calculate the distance from the sensor to the target using initial estimation:

(52) $$d_{i}=\left\|{\theta_{0}}^{\left(1:2\right)}-x_{i}\right\|$$

Construct a diagonal weighted matrix:

(53) $$B=diag\left(d_{2},\;d_{3},\;d_{4}\right)$$

Update error covariance matrix:

(54) $$\Psi =BQB^{T}$$

Using weighted least squares optimization:

(55) $$\theta_{1}={\left({Ga}^{T}\Psi^{-1}Ga\right)}^{-1}{Ga}^{T}\Psi^{-1}h$$

Obtain an improved estimate: $\theta_{1}={\left[x_{1},\;y_{1},\;r_{1,1}\right]}^{T}$. Then, perform constraint optimization and calculate the covariance matrix:

(56) $${Cov}_{\theta_{1}}={\left({Ga}^{T}\Psi^{-1}Ga\right)}^{-1}$$

Construct a new matrix equation system:

(57) sGaz=sh

where

(58) $$\begin{array}{c} z=\left[\begin{array}{c} x^{2}\\ y^{2} \end{array}\right]\\ sGa=\left[\begin{array}{cc} 1 & 0\\ 0 & 1\\ 1 & 1 \end{array}\right]\\ sh=\left[\begin{array}{c} x_{1}^{2}\\ y_{1}^{2}\\ r_{1,1}^{2} \end{array}\right] \end{array}$$

Calculate the weighted matrix and use constrained least squares to solve

(59) $$sB=diag\left(x_{1},y_{1},r_{1,1}\right)$$

(60) $$s\Psi =4sB{Cov}_{\theta_{1}}sB$$

(61) $$z={\left(s{Ga}^{T}s\Psi^{-1}sGa\right)}^{-1}s{Ga}^{T}s\Psi^{-1}sh$$

The final position estimation coordinates are $\hat{x}=sign\left(x_{1}\right)\sqrt{z_{1}}$, $\hat{y}=sign\left(y_{1}\right)\sqrt{z_{2}}$.

### 5.3. Ground Vibration Signal Localization Based on SWD-STA/LTA-AIC-ITWLS

**Figure 3:**
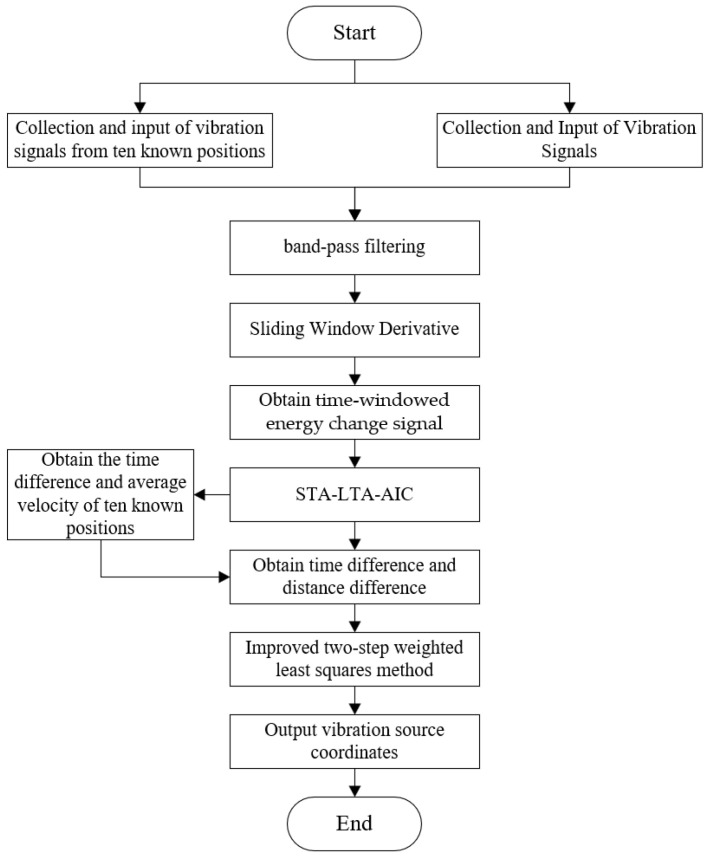
Flowchart of vibration signal localization.

By using the SWD–STA/LTA–AIC–ITWLS algorithm proposed above for positioning calculation, a relatively accurate target position can be obtained. The specific process is shown in Figure 3.

## 6. Experiment and Result Analysis

### 6.1. Configuration Determination of Sensors

The ground vibration signals collected by MEMS accelerometers in outdoor localization are easily reflected and scattered by obstacles such as underground rocks and plant roots, so the deployment area is not easy to be too large. In this experiment, a high-precision MEMS accelerometer developed by the laboratory is used. The sensor parameters include a range of 30 g and a bandwidth of 200 Hz. The sampling rate of each channel is 5 KHz and the sensitive range is a circle with a radius of 5 m.

According to the above conclusion, first test the GDOP value of the sensor edge distance from 0.5 m to 5 m, and then use PSO algorithm to find the sensor coordinates corresponding to the minimum GDOP value in the range of low GDOP value.

**Figure 4:**
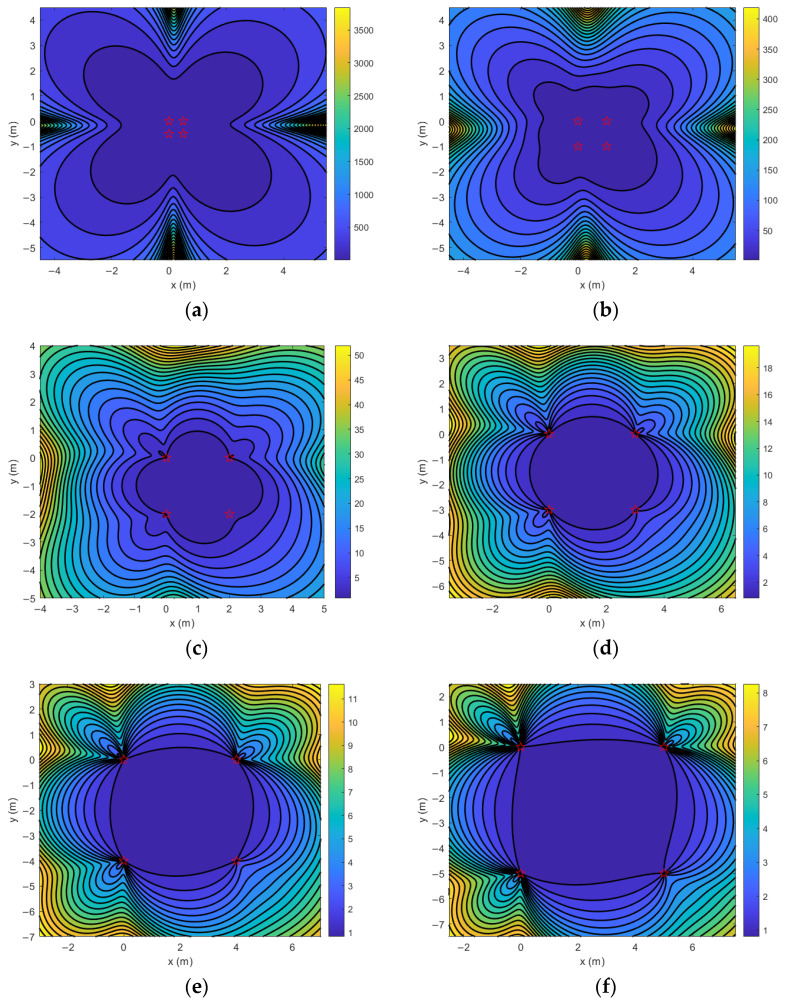
GDOP contour with side length of 0.5 m to 5 m. (**a**) Side length: 0.5 m. (**b**) Side length: 1 m. (**c**) Side length: 2 m. (**d**) Side length: 3 m. (**e**) Side length: 4 m. (**f**) Side length: 5 m. Stars indicate the position of sensors.

**Figure 5:**
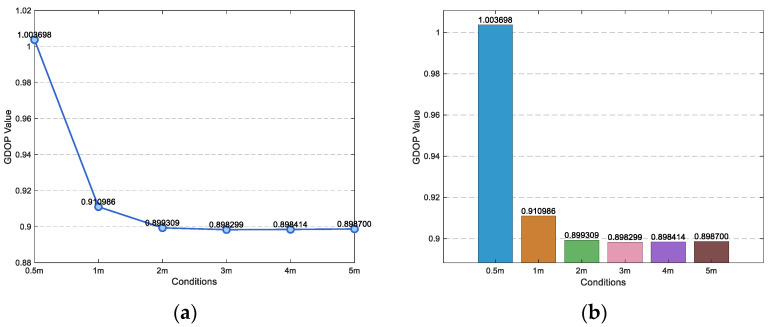
Line chart and bar chart of GDOP value with side length of 0.5 m to 5 m. (**a**) Line chart. (**b**) Bar chart.

As shown in Figure 4 and Figure 5, GDOP values at 0.5 m, 1 m, 2 m, 3 m, 4 m and 5 m margins are 1.003698, 0.910986, 0.899309, 0.898299, 0.898414 and 0.898700, respectively. Therefore, when using PSO algorithm to optimize the minimum value of GDOP, the coordinate range is set to $X\in \left[0,4\right],\;Y\in [-4,0]$, the population size is 50, and the number of iterations is 20.

**Figure 6:**
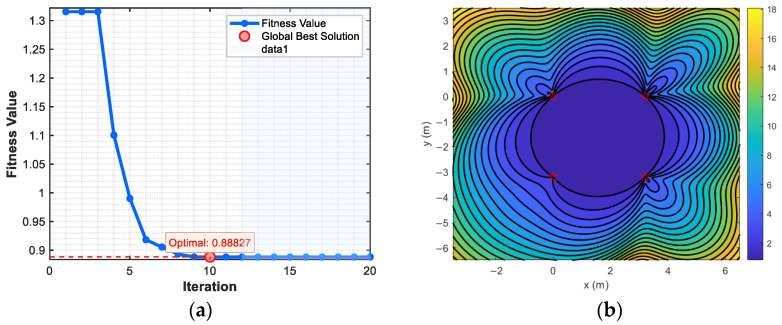
PSO curve and GDOP contour of optimal location. (**a**) PSO curve. (**b**) Side length: 3.2 m. Stars indicate the position of sensors.

The optimization curve of PSO is shown in Figure 6. When 20 iterations are completed, the optimal fitness value is 0.88827, and the optimal parameter is rounded as $S_{1}\left(0,0\right),\;S_{2}\left(3.2,0\right),\;S_{3}\left(3.2,-3.2\right),\;S_{4}\left(0,-3.2\right)$, Therefore, the optimal parameter is taken as the coordinate value of the sensor.

### 6.2. Vibration Signal Localization

#### 6.2.1. On-Site Test Scenario

**Figure 7:**
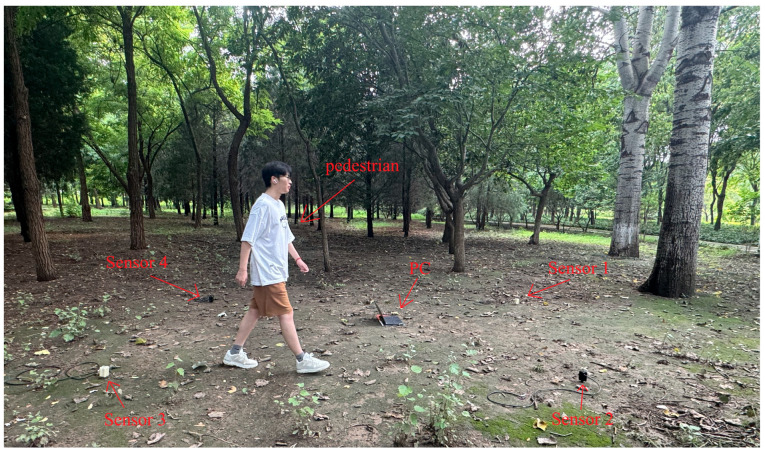
Experimental scenario.

From the above conclusion, it is determined that the configuration of the sensor is a regular quadrangle with a side length of 3.2 m. In the outdoor localization, this area is used as a unit array in the sensor array, and the coordinate position of the vibration source is obtained by collecting the ground vibration signal in this area. The experimental scenario is shown in Figure 7. A surface environment with relatively hard soil was selected and sensors were deployed 10 cm underground to ensure reliable vibration reception. The foot vibration signal is generated by personnel walking, for the convenience of later experimental comparison, the position of personnel walking is marked in advance using differential GPS. The four sensors use the same trigger signal shared with the same data acquisition device to synchronize the sampling clock, and transmit the collected signal to the PC for localization calculation.

#### 6.2.2. Localization Calculation

**Figure 8:**
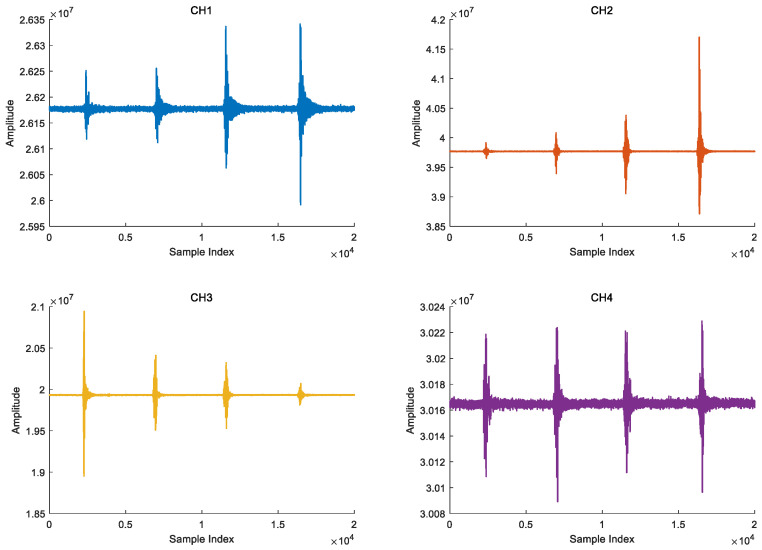
Vibration signals of four channels.

The signal acquisition process is that personnel go from sensor 3 to sensor 2 to simulate the crossing process of personnel invasion. The time domain waveform of the four channels vibration signal is shown in Figure 8.

**Figure 9:**
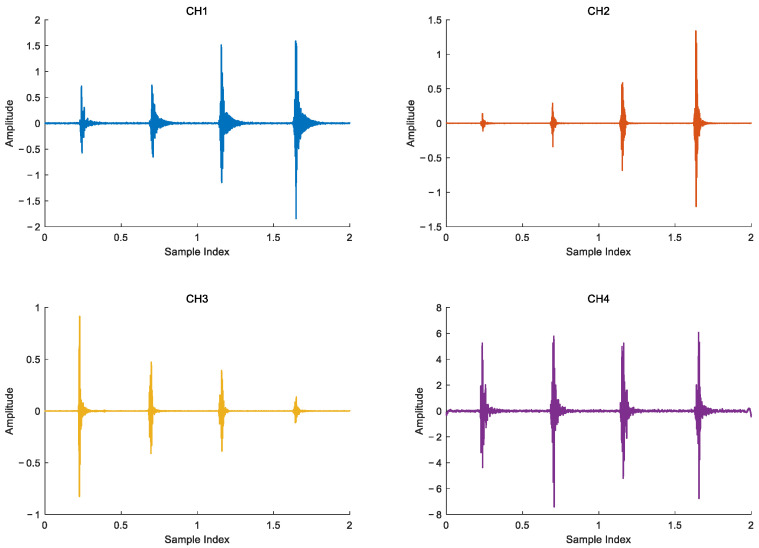
Filtered vibration signal.

Considering the real-time performance of signal processing and the frequency of Rayleigh wave is concentrated near 150 Hz, band-pass filtering is used to process the signal, and the filtering range is [10,200], as shown in Figure 9, the time-domain waveform of the filtered signal is shown.

**Figure 10:**
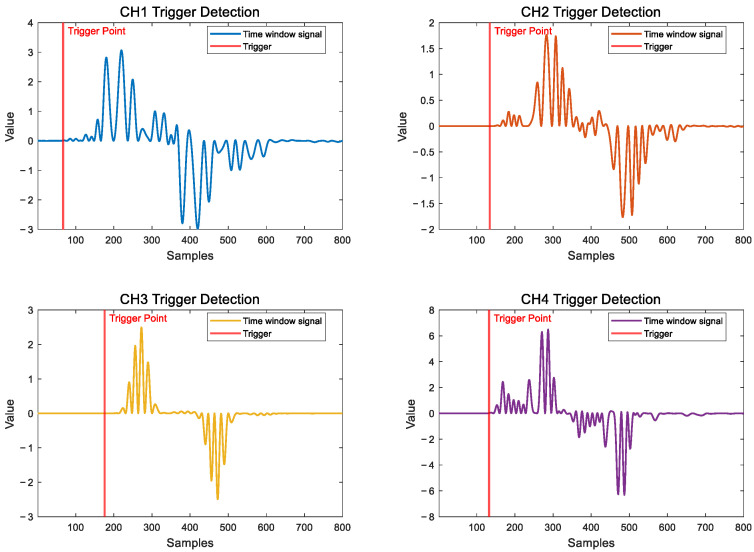
Time window energy change signal and selection of first break.

For the filtered signal, the SWD algorithm proposed in this paper is used to transform the original time domain signal into a time window energy change signal, the size of the sliding window is set to 0.04 ms, and then STA/LTA–AIC algorithm is used to find the first break position of the signal, the window length of STA is 0.02 ms, the window length of LTA is 0.2 ms, and the trigger threshold is 3. Taking the first vibration signal as an example, as shown in Figure 10.

**Figure 11:**
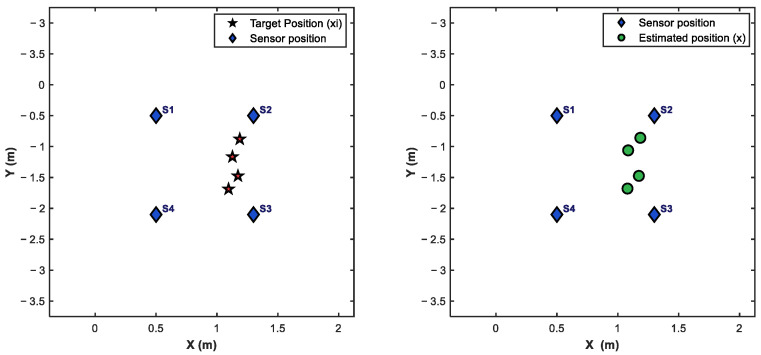
Localization results.

The first break of the output is the time when the signal arrives at the sensor, and the time differences t12, t13, and t14 are obtained by subtracting. The distance difference can be obtained according to R=vt, as shown in Table 1. In order to make the wave velocity closer to the true wave velocity, this experiment measured 9 points within the testing scene range. The coordinates of the 9 points were (0, −0.75), (0, −1.5), (0, −2.25), (1.5, −0.75), (1.5, −1.5), (1.5, −2.25), (3, −0.75), (3, −1.5), (3, −2.25), and obtained the time difference of each point. Based on the coordinates and time difference, the average propagation velocity of the vibration signal at the 9 points can be calculated as 80 m/s, and this average wave velocity is used as the constant wave velocity of the experiment. Due to the small measurement range selected, the attenuation of wave velocity is ignored in this experiment. The final distance differences R12, R13 and R14 are substituted into the two-step weighted least squares algorithm to obtain the target position, as shown in Figure 11 and Table 2.

#### 6.2.3. Evaluation Indicators

In order to analyze the accuracy of the localization algorithm proposed in this paper, the collected vibration source positions are known real coordinates, as shown in Table 3.

The evaluation index selects the average position error and the average direction error, which can better reflect the performance of the localization algorithm. The evaluation index is defined as follows.

(1) Average position error

(62) $$E_{length}=\frac{1}{n}\sum_{1}^{n}\left\|P_{ture}-P_{calc}\right\|$$

where n is the number of targets, $P_{ture}$ is the real coordinate point, and $P_{calc}$ is the calculated coordinate point.

(2) Average direction error

(63) $$E_{direction}=\frac{1}{n}\sum_{1}^{n}\left({cos}^{-1}\left(\frac{\vec{v_{t}}\cdot \vec{v_{c}}}{\left\|\vec{v_{t}}\right\|\cdot \left\|\vec{v_{c}}\right\|}\right)*\frac{180}{\pi }\right)$$

where n is the number of targets, $\vec{v_{t}}$ is the real direction vector, $\vec{v_{c}}$ is the calculated direction vector.

As shown in Table 4, the proposed localization algorithm achieves an average position error of 0.095 m and an average direction error of 0.935 degrees. Compared with the other two traditional localization algorithms, this represents a reduction of 0.637 m in position error and 8.892 degrees in angular error. It is worth noting that all three methods employ the same ITWLS algorithm and STA/LTA-AIC algorithm with identical parameters. They also utilize the same preprocessing steps ([10,200] Hz bandpass filtering) and processed signal length.

## 7. Conclusions

This paper proposes a new fusion localization algorithm: SWD–STA/LTA–AIC–ITWLS. The algorithm is used to calculate the position of the MEMS accelerometer to collect the foot vibration signal. The following conclusions are drawn from the analysis of the actual scene test.

Firstly, this paper uses the PSO algorithm to minimize GDOP to determine the configuration of sensor deployment. Then, the SWD algorithm proposed in this paper is used to preprocess the signal to obtain the time window energy change signal, and the STA/LTA–AIC algorithm is used to obtain the first arrival time of the signal, and calculate the corresponding distance difference. Then, the improved two-step weighted least square method is used to calculate the location, and finally the location of the vibration source is obtained.

The test and analysis results in the actual scene show that the SWD preprocessing method proposed in this paper can better highlight the transient changes in the signal, and can suppress the noise and low-frequency components, which provides a more powerful help for the acquisition of the first break of the signal and the research of the localization algorithm. Compared with the traditional localization algorithm, the proposed fusion algorithm demonstrates significant improvement, decreasing the average position and direction errors by 0.637 m and 8.892 degrees, respectively.

## Figures and Tables

**Table 1:** Calculated distance difference.

Vibration Source	R12 (m)	R13 (m)	R14 (m)
Step 1	−0.9108	−2.5116	−0.9660
Step 2	−0.9520	−4.9280	−0.3920
Step 3	−1.1524	−0.6700	−5.7352
Step 4	−4.8160	0.3360	0.9520

**Table 2:** Calculated coordinates.

Vibration Source	X (m)	Y (m)
Step 1	2.318960	2.318960
Step 2	2.692348	2.692348
Step 3	2.340707	2.340707
Step 4	2.740262	2.740262

**Table 3:** Real coordinates.

Vibration Source	X (m)	Y (m)
Step 1	2.381513	−2.375776
Step 2	2.690587	−1.951677
Step 3	2.508005	−1.330674
Step 4	2.748957	−0.758589

**Table 4:** Average error of different location algorithms.

Localization Algorithm	Average Position Error (m)	Average Direction Error (Degree)
CC-ITWLS	4.214	49.49
STA/LTA–ITWLS	0.732	9.827
SWD–STA/LTA–AIC–ITWLS	0.095	0.935

## Data Availability

Dataset available on request from the authors.
